# Chemical and Physical Characteristics of Edible Films, Based on κ- and ι-Carrageenans with the Addition of Lapacho Tea Extract

**DOI:** 10.3390/foods9030357

**Published:** 2020-03-19

**Authors:** Simona Jancikova, Dani Dordevic, Ewelina Jamroz, Hana Behalova, Bohuslava Tremlova

**Affiliations:** 1 Department of Vegetable Foodstuffs Hygiene and Technology, Faculty of Veterinary Hygiene and Ecology, University of Veterinary and Pharmaceutical Sciences Brno, Palackeho tr. 1946/1, 612 42 Brno, Czech Republic; dordevicd@vfu.cz (D.D.); hana.behalova@gmail.com (H.B.); tremlovab@vfu.cz (B.T.); 2 Department of Technology and Organization of Public Catering, South Ural State University, Lenin Prospect 76, 454080 Chelyabinsk, Russia; 3 Department of Chemistry, University of Agriculture, Balicka Street 122, 30-149 Cracow, Poland; ewelina.jamroz@urk.edu.pl

**Keywords:** antioxidant properties, kappa carrageenan, iota carrageenan, textural properties, lapacho

## Abstract

The aim of the study was to characterize antioxidant and textural property differences of edible films prepared with the addition of lapacho extract (LE). The experimentally produced edible films also contained different carrageenans (ι- and κ-carrageenan). The κ- and ι-carrageenan, glycerol and the different addition of LE (5%, 10%, 20%) were used as ingredients for forming films. The pH and viscosity were measured for film forming solutions (before drying). The following analyses were performed on films: the total polyphenol content (TPC), Ferric Reducing Antioxidant Power (FRAP) and 2-Diphenyl–1-Picrylhydrazyl (DPPH). Optical parameters were analyzed by the determination of UV-Vis spectra. The structure of films was characterized by scanning electron microscopy. The gained results indicated that the use of different gelling agents (ι- and κ-carrageenan) resulted in statistically significant (*p* < 0.05) differences in textural properties (strength and breaking strain) of produced edible films. The highest antioxidant properties and TPC had a κ film with 20% LE (DPPH: 87.63 ± 0.03%; TPC: 233.75 ± 0.104 mg gallic acid/g). According to these results, it can be concluded that edible films with the highest concentrations of added lapacho extract can serve as a good source of antioxidant compounds. Certainly, these properties can be usefully incorporated into the wrapped food commodity.

## 1. Introduction

Different components (polysaccharides, proteins, lipids, etc.) have been used in the preparation of edible packaging materials. Recently, there has been a growing trend of edible packaging materials preparation, due to ecological aspects, and the idea that the additional functional properties could be transferred to wrapped food. On the other side, the history of edible films or packaging goes as far back as the twelfth century, when wax coatings were used to the prolong shelf life of oranges and lemons [1,2]. Edible packaging means the edible thin layer around food, or it represents a physical barrier between the surrounding environment and food. Oxidation is one of the main reasons for food’s short shelf life. Oxidation can be inhibited by the presence of specific antioxidants, that can be found in different plant extracts [3]. The oxidation of food represents the greatest hurdle for the food industry. Oxidation can be stopped by different chemical agents and registered food additives, but there is a high demand from consumers for less processed food and natural substances that can make produced food commodities even more functional [4].

Carrageenans are polysaccharides produced by red seaweeds *Rhodophyceae* [5]. Kappa carrageenan has about 25% to 30% of sulfate content and iota carrageenan about 28 to 30% [6]. Commercial κ- and ι-carrageenans are gel-forming compounds and they are water soluble [5,7,8]. The physicochemical properties of carrageenans are highly affected by their chemical structures (helical structure formation). It was observed that edible films produced with the use of kappa carrageena were clear, with advantageous mechanical and structural properties [9].

The lapacho tea is the inner bark and heartwood of the tree of different botanical names such as *Tabebuia avellanedae* and *Tecoma impetiginosa*. These trees are specific to the area of South America, where lapacho is traditionally used, and this tea is well known for its antitumor properties, mainly due to the presence of polyphenolic compounds [10].

Polyphenolic compounds are common in fruit and vegetables, they are secondary metabolites, and they also have high antioxidant potential and can also work as anti-inflammatory, antimicrobial, antithrombotic and cardio protective compounds [11]. In natural extracts, there can also be a lot of aromatic compounds, which can work as a UV barrier [12]. On the other side, polysaccharides can interact with the polyphenolic compounds, and they can impact the properties of matrices consisting of these compounds. Food commodities represent mainly complex matrixes, in which interactions between the compounds are constantly taking place [13,14].

The aim of the study was to observe differences between edible packaging prepared with κ- and ι-carrageenans and to assess changes of films’ antioxidant properties due to the addition of lapacho extracts.

## 2. Materials and Methods

### 2.1. Materials

The chemicals and solvents used in the analyses were purchased from Sigma Aldrich (St. Louis, MO, USA) and Penta chemicals (Prague, Czech Republic) in ASC purity. Carrageenans were also purchased from Sigma Aldrich. The lapacho tea (*Cortex Tabebuia avell.*) was bought at the local specialized medicinal plants shop (Iva Gežová, Léčivé rostliny, Brno, Czech Republic).

### 2.2. Extract Preparation

Lapacho tea (10 g) was weighed and 50 mL of hot distilled water (100 °C) prepared in the kettle was added. The mixture was extracted for 15 min and then filtrated through Whatman no. 1. The determination of dry matter was done; 25 mL of LE (lapacho extract) was completely dry in the oven (70 °C, 48 h) and the dry matter of LE was 1.91 ± 0.58%.

### 2.3. Film Preparation

We used 0.5 g of ι- or κ-carrageenan for the preparation of films. Moreover, 45 mL of distilled water was added to carrageenan, and then the sample was stirred, until all particles were dissolved. The mixture was heated and then stirred for 10 min at 400 rpm and 70 °C. At the end, 0.25 mL of glycerol was added as plasticizer. In the samples with the addition of lapacho extract, the water was replaced by LE in concentrations 5%, 10% and 20% (κ5% LE, κ10% LE, κ20% LE, ι5% LE, ι10% LE, ι20% LE). The LE was added after 10 min of mixing, and then samples were mixed for another 10 min. The glycerol was added and after 5 min of mixing, the film forming solution was cast in Petri dishes with a diameter of 9 cm and dried in a fume hood for 24 h. The three replicates of each film were prepared. In the text, the word sample is used for films or film forming solutions.

### 2.4. pH

The pH of the film forming solutions was evaluated using a pH meter (GRYF 259, Havlickuv Brod, Czech Republic).

### 2.5. Viscosity

The viscosity of the film forming solution immediately after preparation (temperature of solution = 60 °C) was measured by Haake viscotester 7 plus (Thermo Electron, Karlsruhe, Germany). The measuring was repeated in triplicate and viscosity was measured in MPa·s and percentage.

### 2.6. Fourier Transform Infrared Spectra (FTIR)

A FTIR analysis of carrageenan films and their composite with lapacho extracts was done by Fourier-transform infrared spectrophotometer (MATTSON 3000 FT-IR, Madison, WI, USA). The FTIR spectra were recorded in the wavelength range of 4000–400 cm^−1^, with 4 cm^−1^ resolution.

### 2.7. Textural Analysis

An analysis of the textural properties strength (MPa), breaking strain, elongation at break (%) and toughness (MJ/m^3^) was performed using a texturometer TA.XT plus (Godalming, UK). The measurements were done according to the ASTM International test method—ASTM D882-02. Each film was cut to dimensions 5 × 1 cm and tested with an initial grip separation of 50 mm and test speed 8.3 mm/s.

### 2.8. Thickness

The thickness of films was measured by micrometer Mitutoyo M310-25 (Kawasaki, Japan) at 3 different places (2 films from each type).

### 2.9. Water Content and Solubility

The 3 squares (2 × 2 cm) were cut from the films and weighed (W1), then the films were put in the Ecocell 55 (105 °C) oven for 2 h and then weighed again (W2). The squares were put in the beaker containing 25 mL of distilled water, and after 24 h at laboratory temperature, the films were dried and weighed (W3). Water content and solubility were calculated according to the following formulas:

Water content (%) = [(W1−W2)/W1] × 100

Solubility (%) = [(W2−W3)/W2] × 100

### 2.10. Ferric Reducing Antioxidant Power (FRAP)

FRAP, described by Behbahani et al. [15] with slight modifications, was evaluated with the use of TPTZ (2,4,6-Tris(2-pyridyl)-s-triazine), acetic buffer and FeCl_3_. Then, 0.1 g of sample was weighed and extracted in 75% methanol for 30 min in an ultrasound water bath at laboratory temperature. The extract was filtrated and mixed (same as the 100% LE and diluted LE in concentrations 5%, 10% and 20%) with a reagent solution (5 mL of TPTZ + 5 mL of FeCl_3_ + 25 mL of acetic buffer). After 8 min of incubation at laboratory temperature, the absorbance was measured at 593 nm by spectrophotometer (CE7210 DIET-QUEST, Cambridge, England). The same procedure was used for the preparation of the blank sample; the sample was replaced with distilled water. Trolox was used as a standard.

### 2.11. 2-Diphenyl−1-Picrylhydrazyl (DPPH) 

DPPH scavenging activity was prepared according to the method described by Sivarooban et al. [16] with slight modifications. 0.1 g of sample was weighed and extracted with 20 mL of ethanol for 30 min in an ultrasound water bath at the laboratory temperature. The extract was filtrated and 3 mL of film extract (same as the 100% LE and diluted LE in concentrations 5, 10 and 20%) was mixed with 1 mL of 0.1mM DPPH solution in ethanol, the sample was incubated in the dark at laboratory temperature. The absorbance was measured at 517 nm by spectrophotometer (CE7210 DIET-QUEST, Cambridge, England). Ethanol was used as a blank sample. The DPPH scavenging activity was calculated as follows:

DPPH scavenging activity [%] = [(Abs_DPPH_ − Abs_sample_)/Abs_DPPH_) × 100

### 2.12. Total Polyphenols Content (TPC)

TPC was prepared according to Tomadoni et al. [17], with slight modification. Then, 1 g of sample (or 1 mL 100% LE and diluted LE in concentrations 5, 10 and 20%) was weighed and 40 mL of distilled water was added and mixed for 10 min at the laboratory temperature, then the sample was mixed with Folin–Ciocalteau (1:10) and 7.5% Na_2_CO_3_. The mixture was incubated for 30 min at laboratory temperature and the absorbance was measured at 765 nm by spectrophotometer (CE7210 DIET-QUEST, Cambridge, England). The gallic acid was used as a standard.

### 2.13. UV-Vis Spectra

The measuring of UV-Vis was done by spectrophotometer (CE7210 DIET-QUEST, Cambridge, England) at wavelengths from 200 to 600 nm. The transmittance (%) was calculated for the following wavelengths: 254, 280 (UV-C), 300 (UV-B), 325 (UV-A) and 600 (Vis region).

### 2.14. Scanning Electron Microscopy (SEM)

The films were cut using scissors. The microstructure/surface of prepared films was measured by a scanning electron microscope MIRA3 TESCAN at accelerating voltage 5.0 kV. Each film sample was scanned at 3 different areas.

### 2.15. Statistical Analysis

Statistical significance at *p* < 0.05 was evaluated by a one-way ANOVA analysis of variance, and parametric Tukey post-hoc test (in the case when Levene’s test showed equal variances *p* > 0.05) and a nonparametric Games–Howel post hoc test (in the case when Levene’s test showed unequal variances *p* < 0.05) for finding differences within groups. The overall differences among samples were checked by principal component analysis (PCA). SPSS 20 statistical software (IBM Corporation) was used for all applied analysis. 

## 3. Results and Discussion

The experimentally produced carrageenan films were transparent and homogenous. Regardless of the type of carrageenan used, the films became brown and less transparent as the extract addition increased.

### 3.1. Viscosity and pH of Film Forming Solution

The results of pH and viscosity are summarized in Table 1. The pH of film forming solutions decreased with the addition of lapacho extract, of which the pH was 4.23 ± 0.01. The significant differences (*p* < 0.05) were found between all samples with κ-carrageenan. In addition, significant (*p* < 0.05) changes between ι5%LE, ι10% LE and ι20% LE solutions were noted too. The natural extracts are the source of flavonols, flavonoids, anthocyanins, and also phenolic acids. The lapacho consists of caffeic, protocatechuic, p-coumaric, ferulic, and also syringic acid [18].The phenolic acids cause the lower pH of natural plant extracts [19,20].

Significant differences between samples in the measuring of viscosity were not found, and except κC and κ20%LE, the viscosity was expressed in percentage. A downward trend was observed, meaning that the addition of extracts can decrease the viscosity of film forming solution.

### 3.2. Fourier Transform Infrared Spectra

**Figure 1:**
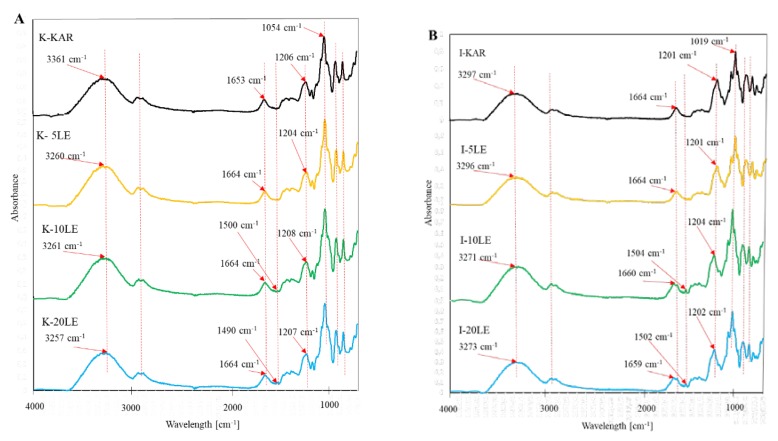
(**A**) i-carrageenan films, (**B**) k-carrageenan films. FT-IR spectra. κC–κ-carrageenan control film, κ5% LE—κ-carrageenan with 5% of lapacho extract, κ10% LE—κ-carrageenan with 10% of lapacho extract, κ20% LE–κ-carrageenan with 20% of lapacho extract, ιC—ι-carrageenan control film, ι5% LE—ι-carrageenan with 5% of lapacho extract, ι10% LE—ι-carrageenan with 10 % of lapacho extract, ι20% LE–ι-carrageenan with 20% of lapacho extract.

The FTIR spectra of i-carrageenan, k-carrageenan films and their biocomposite films with lapacho extracts in different concentrations are shown in Figure 1A,B.

The spectrum of k-carrageenan (Figure 1) showed characteristic peaks in the absorption band 4000–400 cm^−1^. The peaks at 900 cm^−1^ and 848 cm^−1^ are assigned to 3,6-anhydro-D-galactose and galactose-4-sulfate, respectively [3]. In κ-carrageenan films, the characteristic peaks are at 3361 cm^−1^ (OH stretching vibration), 29,746 cm^−1^ (CH stretching vibration) and 1206 cm^−1^ (ester sulfate O=S=O symmetric vibration) [21].

The ι-carrageenan consists of −O−SO_3_ stretching vibration at D-galactose-4-sulfate (G4S) and D-galactose-2-sulfate (DA2S) (891 cm^−1^ and 842 cm^−1^), C−O−C of 3,6-anhydro-D-galactose (930 cm^−1^), C−O stretch (1019 cm^−1^), ester sulfate O=S=O symmetric vibration (1201 cm^−1^), C−O bridge stretch (1382 cm^−1^), water deformation (1664 cm^−1^), C−H stretch (2907 cm^−1^) and O−H stretch (3297 cm^−1^), respectively [22].

After the addition of the lapacho extract, the appearance of a new peaks from the aromatic ring (C=C) of β-lapachone at wavenumbers of ~1490 cm^−1^ (K-KAR) and ~1502 cm^−1^ (I-KAR) were observed [23].

In the case of ι-carrageenan films, the addition of lapacho extract causes the appearance of an additional peak, at a wavelength of ~1659 cm^−1^, which may indicate the presence of the carbonyl groups (C=O) of β-lapachone. The changes in the appearance of the FT-IR spectrum in k- and ι-carrageenan films can be observed in the absorption band 1550−1300 cm^−1^, which may be due to the presence of methyl (-CH_3_) and methylene (-CH_2_-) groups derived from lapachol [23].

These observations may suggest that there are interactions between carrageenans and lapacho extract.

### 3.3. Textural Analysis

The results of textural properties are presented in Table 2. The type of carrageenan used in preparing films, significantly (*p* < 0.05) affected the strength parameter. Moreover, the addition of LE caused an increase in strength. κ5% LE and κ10% LE were significantly different (*p* < 0.05) in comparison with κC. However, in the case of ι-type of films, the statistical differences were not observed. The texture and tensile strength can be affected by the pH of the film-forming solution. [24,25]. According to the literature data, the strongest films are consisting κ-carrageenan, because it has the lowest amount of sulfate groups and a negative charge [26].

The breaking strain parameter increased when LE was added and statistically significant (*p* < 0.05) differences were observed between κ10% LE and κ20% LE. Significant differences (*p* < 0.05) between κ samples and ι samples were found. This can be explained by the presence of natural extract (LE), which contains a high amount of phenolic compounds and new interactions can be formed between these polyphenols and polysaccharides, such as the hydrogen bonds [13].

The tensile strength was too low, but braking strain is very high compared with the properties of films reported in the literature [26].

### 3.4. Thickness, Water Content and Solubility

Table 3 shows the experimental results of thickness, water content and solubility. It was observed that the thickness was higher when LE extract was added in higher concentration. The thickness can be higher due to extracts addition [27,28]. Thickness in films containing κ-carrageenan increased by up to ~26%, but significant differences (*p* < 0.05) were not observed. In samples containing ι-carrageenan, the thickness was increased by up to ~ 26% and between ιC and ι20%, LE was observed as having a significant difference (*p* < 0.05). The use of different carrageenans (ι- and κ) did not influence films’ thickness. According to the literature data, films are defined by a thickness of less than 100 μm and usually they are used for wrapping the product, overwrapping packaging or to make sachets, bags etc. [29]. The thickness of edible films and coatings is usually less than 0.3 mm, so the films produced during this research are in this limit. When the packaged product is eaten together with edible films, it is better when the thickness of the film or coating is as small as possible, so that the packaging does not impact the sensory properties as well as the appearance of the foodstuff [26].

The water content of films did not differ significantly within samples prepared with κ-carrageenan same as within samples prepared ι-carrageenan. The results observed for κC, κ5% LE and κ10% LE were significantly different (*p* < 0.05) in comparison with ιC. It means that the use of different carrageenan had an impact on water content. The samples containing ι carrageenan had lower water content than samples containing κ carrageenan. A different addition of lapacho extract influenced the water content; the water content decreased with higher addition of lapacho extract. The similar results were found in the previous study by Liu et al. [14]; the addition of mulberry polyphenolic extract reduced water content in tested films. The reducing water content, with the addition of natural extracts, can be explained by the reactions of phenolic hydroxyl groups in natural extracts with hydroxyl groups in carrageenan and these intramolecular interactions (for example hydrogen bonds) can impact the interaction between carrageenan and water [14].

The solubility of all films was 100%. The finding is in accordance with previous studies, where the good solubility of carrageenans was also found, and they were marked as hydrophilic colloids [5,8].

### 3.5. Antioxidant Properties of Films

The results of antioxidant properties of edible films are presented in Table 4. The total polyphenols content in LE was 30.53 ± 0.09 mg gallic acid/mL. In samples without the addition of LE, there were also small amounts of polyphenolic compounds in κC (8.11 ± 0.20 mg/g) and in ιC (4.32 ± 0.10 mg/g). Previous research by de Souza et al. [30] found out that polysaccharides from red algae (such as carrageenans), have an antioxidant activity and this activity is correlated with the amount of sulphated groups. The higher amount of sulphate is usually in iota carrageenan, so it should have higher antioxidant properties [4,27]. The addition of LE resulted in a TPC increase and the highest was in κ20% LE (233.75 ± 0.104 mg gallic acid/g). Significant differences were found (*p* < 0.05) between all samples except κ5%LE; meaning that the addition of LE has a high impact on TPC, due to the fact that natural extracts contain a lot of polyphenolic compounds [11]. Polyphenols can also react with polysaccharides, where the most common interactions are hydrogen bonds and hydrophobic interactions [31].

A FRAP analysis showed that LE had 7.04 ± 0.05 µmol Trolox/mL. The highest amount was found in κ20% LE (38.78 ± 0.15 µmol Trolox/g) and ι20% LE (46.69 ± 0.18 µmol Trolox/g); between these samples, a statistically significant difference (*p* < 0.05) was observed. The significant (*p* < 0.05) difference was found among all investigated samples; only ιC and κC did not differ significantly.

DPPH scavenging activity results showed that the highest antioxidant activity had κ20% LE (87.63 ± 0.03%) and ι20% LE (69.13 ± 0.12%), and had the same trend; higher LE addition resulted in increased antioxidant activity.

TPC, FRAP and DPPH measurements indicated that the higher addition of LE resulted in increased TPC among κ samples, but FRAP and DPPH were lower. It can be explained by the fact that not every polyphenolic compound has antioxidant properties. When no LE was added, ιC had lower (*p* < 0.05) TPC than κC, though in the case of FRAP the amount of Trolox was higher, but with no significant difference (*p* < 0.05), and in DPPH analysis no differences were observed between ιC and κC. The differences could also be caused by using different extraction solutions for each method (TPC, FRAP, DPPH).

In the literature, it can be found that TPC correlates with antioxidant activity, but it was also found that the correlation is not clear in each case. Wong et al. [32] found that the correlation of total polyphenols content with DPPH and FRAP was only detected partially. Another explanation is that FRAP and DPPH measuring include different conditions. FRAP method is performed in a very low pH value (about 3.6) and also the mechanism is different. DPPH works as follows: the antioxidant compound reacts by the reduction of the radical, and decreased absorbance is measured. On the other hand, the FRAP assay can indicate new formed ferrous ions, and increasing absorbance is measured [33].

The DPPH radical scavenging activity was also affected by pH, because pH is lower in the presence of phenolic acids; the acid can donate the hydrogen to the DPPH radical, where the nitrogen atom is reduced, so the product loses the violet color and a lower absorbance of solution is measured [34].

In previous studies, it was found that in films with the addition of 20% rosemary extract TPC was 3.87 ± 0.0 mg GAE/g of dried sample, when an aqueous extract of fresh rosemary was added and 6.79 ± 0.06 mg GAE/g of dried samples, when an aqueous extract of dried rosemary was added [27]. The comparison of FRAP and DPPH showed that dried rosemary extract is a better source of antioxidant compounds than lapacho tea—the DPP of films with the addition of 20% of rosemary extracts showed DPPH 87.84 ± 0.07% and FRAP 207.08 ± 1.30 µmol Trolox/g [27]. In films with tea polyphenols, the maximum TPC (above 160 mg/g sample) was found in the film with 40% of tea polyphenols [35]. The results for all ι-LE films and κ-LE films, containing up to 10% LE, were in the scope of the results found in the literature, but κ20% LE was higher than results from previous studies. The presence of antioxidants is important, because the film can work as the carrier of these compounds and can improve the shelf life of fresh as well as minimally processed fruits and vegetables [36].

Table 5 presents the results of TPC, FRAP and DPPH in lapacho extracts in concentrations added to the prepared films (5%, 10% and 20%) and for 100% lapacho extract. When the results are compared with the antioxidant properties of films, the value found in films is significantly higher. It has to be stressed that there is an impact of carrageenan addition and interactions between carrageenan and polyphenolic compounds.

The DPPH values decreased with the higher amounts of LE extracts. This observation is the opposite from the finding that with extract concentrations the total polyphenol content and FRAP increased, due to a higher amount of antioxidant compounds. The results of DPPH are probably affected by the color of the extract, due to the yellow-orange color. DPPH in the presence of antioxidant compounds is yellowish colored. It means that orange color can affect the measured absorption. The 100% LE absorption (~0.95) was almost five times higher than the absorption of the DPPH solution (~0.28).

The samples were not affected by the color of the extract, because just 0.1 g of films were used for the extraction of antioxidant compounds.

### 3.6. UV-Vis Spectra and Transmittance

**Figure 2:**
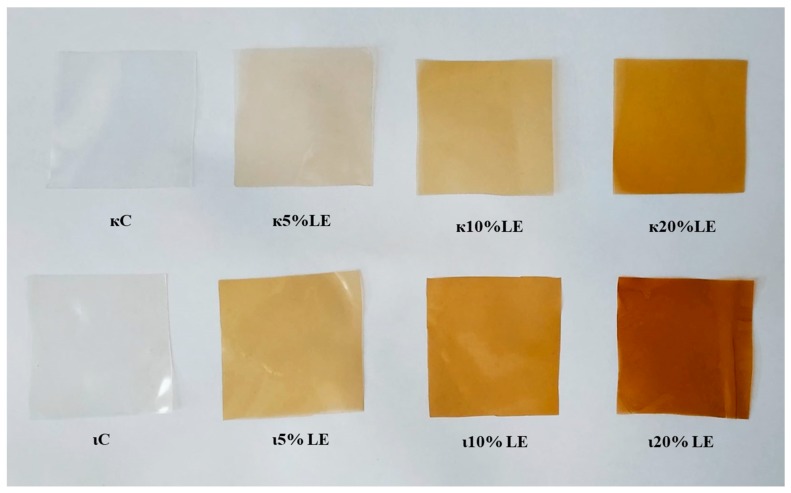
The appearance of active films. κC – κ-carrageenan control film, κ5% LE—κ-carrageenan with 5% of lapacho extract, κ10% LE—κ-carrageenan with 10% of lapacho extract, κ20% LE—κ-carrageenan with 20% of lapacho extract, ιC—ι-carrageenan control film, ι5% LE—ι-carrageenan with 5% of lapacho extract, ι10% LE—ι-carrageenan with 10% of lapacho extract, ι20% LE—ι-carrageenan with 20% of lapacho extract.

The appearance of films is shown in Figure 2. Natural pigments could cause the differences in color appearance of films, so the anthocyanins in lapacho tea are pH sensitive [37,38]. When the color appearance of films is compared to the pH of the film forming solution, it can be said that color is affected by the amount of the extract, but also by pH. So, the color differences between ι- and κ-carrageenan samples are caused by the pH of the film forming solution.

**Figure 3:**
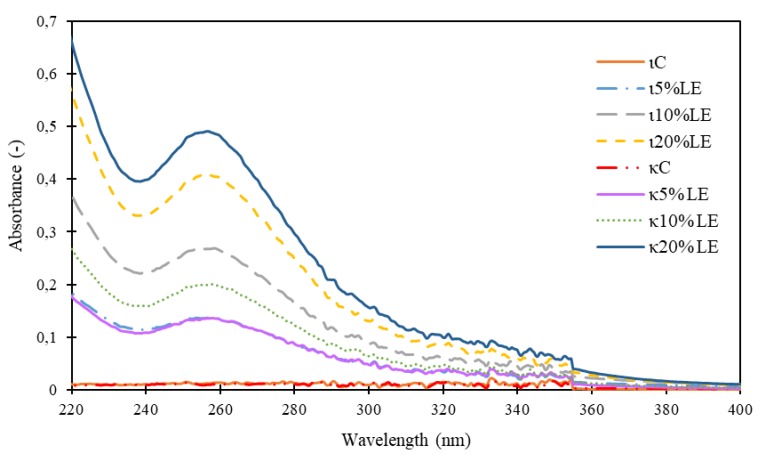
UV-Vis spectra. κC–κ-carrageenan control film, κ5% LE—κ-carrageenan with 5% of lapacho extract, κ10% LE—κ-carrageenan with 10% of lapacho extract, κ20% LE—κ-carrageenan with 20% of lapacho extract, ιC—ι-carrageenan control film, ι5% LE—ι-carrageenan with 5% of lapacho extract, ι10% LE—ι-carrageenan with 10% of lapacho extract, ι20% LE—ι-carrageenan with 20% of lapacho extract.

The UV-Vis spectra of experimentally produced films are shown in Figure 3. It was observed that the addition of LE can impact the UV radiation. The transmittance for certain wavelengths was calculated and in the case of transmittance in UV-C region, the films with higher amount of LE absorbed the highest amount of radiation; only a little amount permeate through this. In the UV-B and UV-A region, the films with 20% LE absorbed the highest amount of radiation, though the differences were not as high as in the UV-C region. The protection against UV radiation is an important property of packaging, since UV radiation can damage the compounds present in food such as vitamins, carotenes or unsaturated fatty acids [39].

The results of transmittance are presented in Table 6. The transmittance indicates how much light can get through the material [40]. The results stressed that the addition of LE extract had an impact on light transmission. Significant differences were found (*p* < 0.05) between almost all samples, meaning that the addition of different amounts of lapacho extract to films can significantly (*p* < 0.05) impact the light transmission. The Vis region reached almost 100% in all samples, no matter if it was with the addition of LE or not. The light transmittance was reduced with the decreasing wavelength and with the addition of LE. The lowest values were found in κ20% LE (32.60 ± 0.78%) and ι20% LE (39.36 ± 0.12), emphasizing that these films serve as the best prevention against UV radiation.

The UV protection of natural extracts is caused by the presence of aromatic compounds [12]. These types of films can be used as a UV protector, because a lot of unwanted reactions occurring in foodstuffs are caused by UV radiation. The typical susceptible foodstuffs are ham and drinks [28]. The most common reactions are protein fragmentation, carbohydrate cross linking and the peroxidation of unsaturated fatty acids [41].

### 3.7. Scanning Electron Microscopy

Scanning electron microscopy (Table 7) shows the layer structure and surface smoothness and the thickness of edible films [26]. The cross-section of prepared edible films consisted of small layers. The cross-section was more compact in the case of κ-carrageenan than in ι-carrageenan, especially in ιC and ι5%LE. ι10% LE and ι20% LE were compact in a similar way as the samples consisting of κ-carrageenan. The difference between the appearances of cross-sections can be caused by the scissors used in sample preparation.

### 3.8. Principal Component Analysis (PCA)

**Figure 4:**
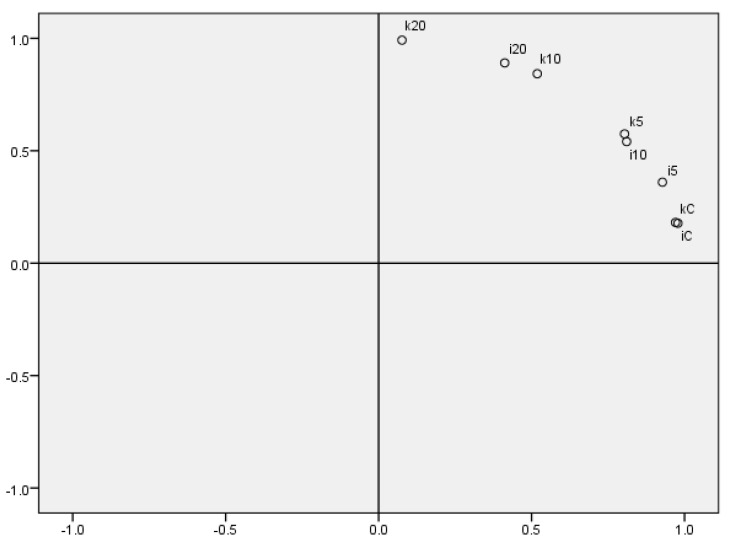
Principal component analysis of researched films. κC–κ-carrageenan control film, κ5% LE—κ-carrageenan with 5% of lapacho extract, κ10% LE—κ-carrageenan with 10% of lapacho extract, κ20% LE—κ-carrageenan with 20% of lapacho extract, ιC—ι-carrageenan control film, ι5% LE—ι-carrageenan with 5% of lapacho extract, ι10% LE—ι-carrageenan with 10% of lapacho extract, ι20% LE—ι-carrageenan with 20% of lapacho extract.

PCA (Figure 4) summarized all the results for each sample. The first principal component explained more than 80 % of the total variation (κC, ιC, ι5, ι10, κ5); with the second more than 97% (ι20, κ10), and the first three principal components (κ20) explained more than 99% of the total variation. It can be seen that when different carrageenans were used, the higher amount of LE in ι samples could impact the properties of samples, which are similar to κ samples consisting of lower amount of LE. The most differing samples were κ20%LE; as was found in previous results, this sample had the highest total polyphenol content and DPPH scavenging activity; these samples also had the potential to work as a UV protector. However, this property should be checked by further detailed examination.

## 4. Conclusions

The aim of this research was to examine the properties of edible films consisting of ι- and κ-carrageenans, to find out the differences between the use of these two carrageenans and to know how films’ properties can be improved by the addition of lapacho extract. This study emphasized the differences between ι- and κ- films, especially the different textural properties. The impact of LE addition was the highest in the case of 20% LE addition, because there was the highest amount of polyphenolic compounds, so the films had higher antioxidant activity, UV protecting properties and the water content was also the lowest. PCA separated the sample κ20%LE, which has the most favorable results between all experimentally produced samples. Therefore, the results of the study will certainly serve as a good source of information for further edible packaging formulae, same as for the possible application on suitable types of food commodities. The experimentally produced packaging can serve as a good source of antioxidant compounds. The films could potentially work as the active films for fruits and vegetables and improve their shelf-life. It is certain that further study will give a clearer picture about the properties of these kinds of edible films and packaging, and about their application on foodstuffs.

## Figures and Tables

**Table 1:** pH and viscosity of film forming solutions.

Sample	pH (-)	Viscosity (%)	Viscosity (mPa)
κC.	9.21 ± 0.03 ^A^	9.33 ± 0.29 ^A^	90.00 ± 0.00
κ5% LE	7.39 ± 0.07 ^B^	8.33 ± 0.29	80.00 ± 0.00
κ10% LE	6.59 ± 0.07 ^C^	8.00 ± 0.00	80.00 ± 0.00
κ20% LE	5.62 ± 0.13 ^D^	7.50 ± 0.00 ^B^	70.00 ± 0.00
ιC	9.45 ± 0.11	14.00 ± 5.27	136.67 ± 50.33
ι5% LE	8.27 ± 0.09 ^E^	10.67 ± 2.75	103.33 ± 25.17
ι10% LE	7.38 ± 0.04 ^B^	12.50 ± 2.50	123.33 ± 25.17
ι20% LE	6.19 ± 0.03 ^D^	12.50 ± 2.78	123.33 ± 30.55

Different letters in the same column indicate statistically significant (*p* < 0.05) differences. κC – κ-carrageenan control film, κ5% LE—κ-carrageenan with 5% of lapacho extract, κ10% LE—κ-carrageenan with 10% of lapacho extract, κ20% LE—κ-carrageenan with 20 % of lapacho extract, ιC—ι-carrageenan control film, ι5% LE—ι-carrageenan with 5% of lapacho extract, ι10% LE—ι-carrageenan with 10% of lapacho extract, ι20% LE—ι-carrageenan with 20 % of lapacho extract.

**Table 2:** Textural properties of edible films.

Sample	Strength (MPa)	Breaking Strain (%)
κC	0.35 ± 0.03 ^A^	72.0 ± 1.6 ^A^
κ5% LE	0.42 ± 0.02 ^B^	77.6 ± 1.6 ^AB^
κ10% LE	0.41 ± 0.03 ^B^	79.0 ± 1.7 ^B^
κ20% LE	0.40 ± 0.02 ^AB^	79.1 ± 2.1 ^B^
ιC	0.15 ± 0.00 ^C^	99.1 ± 0.5 ^C^
ι5% LE	0.16 ± 0.01 ^C^	97.2 ± 3.3 ^C^
ι10% LE	0.15 ± 0.01 ^C^	113.5 ± 4.6
ι20% LE	0.14 ± 0.01 ^C^	90.2 ± 7.9

Different letters in the same column indicate statistically significant (*p* < 0.05) differences. κC – κ-carrageenan control film, κ5% LE—κ-carrageenan with 5% of lapacho extract, κ10% LE—κ-carrageenan with 10% of lapacho extract, κ20% LE—κ-carrageenan with 20% of lapacho extract, ιC—ι-carrageenan control film, ι5% LE—ι-carrageenan with 5% of lapacho extract, ι10% LE—ι-carrageenan with 10% of lapacho extract, ι20% LE—ι-carrageenan with 20% of lapacho extract.

**Table 3:** Results of thickness, water content and solubility of edible packaging.

Sample	Thickness (mm)	Water Content (%)	Solubility (%)
**κC**	0.088 ± 0.007 ^A^	18.16 ± 1.11 ^A^	100 ± 0
**κ5% LE**	0.102 ± 0.016	18.78 ± 1.06 ^A^	100 ± 0
**κ10% LE**	0.095 ± 0.013	16.92 ± 1.26 ^A^	100 ± 0
**κ20% LE**	0.112 ± 0.029	15.09 ± 0.44	100 ± 0
**ιC**	0.088 ± 0.005 ^A^	15.86 ± 1.63	100 ± 0
**ι5% LE**	0.088 ± 0.002 ^A^	15.79 ± 2.03	100 ± 0
**ι10% LE**	0.101 ± 0.013	14.08 ± 3.30	100 ± 0
**ι20% LE**	0.111 ± 0.007 ^B^	11.67 ± 2.26 ^B^	100 ± 0

Different letters in the same column indicate statistically significant (*p* < 0.05) differences. κC – κ-carrageenan control film, κ5% LE—κ-carrageenan with 5% of lapacho extract, κ10% LE—κ-carrageenan with 10% of lapacho extract, κ20% LE—κ-carrageenan with 20% of lapacho extract, ιC—ι-carrageenan control film, ι5% LE—ι-carrageenan with 5% of lapacho extract, ι10% LE—ι-carrageenan with 10% of lapacho extract, ι20% LE—ι-carrageenan with 20% of lapacho extract.

**Table 4:** Result of TPC, FRAP and DPPH in researched films.

Sample	TPC (mg Gallic Acid/g of Sample)	FRAP (µmol Trolox/g of Sample)	DPPH (%)
**κC**	8.11 ± 0.20 ^A^	0.31 ± 0.10 ^A^	n.d.
**κ5% LE**	71.51 ± 0.14	12.51 ± 0.11 ^B^	33.60 ± 0.55 ^B^
**κ10% LE**	128.71 ± 0.231 ^B^	25.38 ± 0.11 ^C^	69.62 ± 0.10 ^C^
**κ20% LE**	233.75 ± 0.104 ^C^	38.78 ± 0.15 ^D^	87.63 ± 0.03 ^D^
**ιC**	4.32 ± 0.10 ^D^	0.53 ± 0.03 ^A^	n.d.
**ι5% LE**	37.79 ± 0.14 ^E^	7.53 ± 0.01 ^E^	n.d.
**ι10% LE**	61.16 ± 4.89 ^E^	14.71 ± 0.11 ^G^	34.46 ± 0.42 ^B^
**ι20% LE**	147.73 ± 0.26 ^F^	46.69 ± 0.18 ^H^	69.13 ± 0.12 ^E^

Different letters in the same column indicate statistically significant (*p* < 0.05) differences. κC–κ-carrageenan control film, κ5% LE—κ-carrageenan with 5% of lapacho extract, κ10% LE—κ-carrageenan with 10% of lapacho extract, κ20% LE—κ-carrageenan with 20% of lapacho extract, ιC—ι-carrageenan control film, ι5% LE—ι-carrageenan with 5% of lapacho extract, ι10% LE—ι-carrageenan with 10% of lapacho extract, ι20% LE—ι-carrageenan with 20% of lapacho extract.

**Table 5:** Result of TPC, FRAP and DPPH in lapacho extracts.

Sample	TPC (mg Gallic Acid/mL of LE Extract)	FRAP (µmol Trolox/mL of LE Extract)	DPPH (%)
**5% LE**	1.010 ± 0.002 ^A^	0.086 ± 0.001 ^A^	84.36 ± 0.04 ^A^
**10% LE**	1.919 ± 0.002 ^B^	0.274 ± 0.002 ^B^	77.43 ± 0.06 ^B^
**20% LE**	3.583 ± 0.001 ^C^	0.596 ± 0.003 ^C^	65.18 ± 0.22 ^C^
**100% LE**	30.528 ± 0.090 ^D^	7.044 ± 0.048 ^D^	o.d.l.

Different letters in the same column indicate statistically significant (*p* < 0.05) differences, o.d.l.: over detection limit.

**Table 6:** Results of transmittance expressed in %.

Sample	T254 (UV-C)	T280 (UV-C)	T300 (UV-B)	T325 (UV-A)	T600 (Vis Region)
κC	97.74 ± 0.07 ^A^	97.53 ± 0.49 ^A^	97.79 ± 0.52 ^A^	97.68 ± 0.67 ^A^	99.87 ± 0.04 ^AC^
κ5% LE	73.69 ± 0.43 ^B^	81.86 ± 0.32 ^B^	89.70 ± 0.34 ^B^	92.97 ± 0.38 ^B^	100.00 ± 0.00 ^A^
κ10% LE	63.43 ± 1.88 ^C^	75.53 ± 1.33 ^B^	86.38 ± 0.64 ^C^	91.46 ± 1.03 ^BD^	99.96 ± 0.18 ^A^
κ20% LE	32.60 ± 0.78 ^D^	50.52 ± 1.03 ^C^	69.83 ± 0.97 ^D^	80.57 ± 0.59 ^C^	99.55 ± 0.16 ^CD^
ιC	97.35 ± 0.26 ^A^	97.03 ± 0.25 ^A^	97.81 ± 0.21 ^A^	97.30 ± 0.56 ^A^	99.89 ± 0.10 ^A^
ι5% LE	73.07 ± 0.15 ^BC^	81.85 ± 0.17 ^B^	89.57 ± 0.42 ^B^	92.98 ± 0.41 ^B^	99.53 ± 0.09 ^D^
ι10% LE	53.99 ± 0.12 ^C^	68.46 ± 0.22 ^D^	81.58 ± 0.41 ^E^	87.40 ± 0.31 ^D^	98.98 ± 0.10 ^B^
ι20% LE	39.36 ± 0.12 ^E^	56.33 ± 0.39 ^E^	73.99 ± 0.47 ^F^	83.46 ± 0.08 ^C^	99.44 ± 0.15 ^D^

Different letters in the same column indicate statistically significant (*p* < 0.05) differences. κC–κ-carrageenan control film, κ5% LE—κ-carrageenan with 5% of lapacho extract, κ10% LE—κ-carrageenan with 10% of lapacho extract, κ20% LE—κ-carrageenan with 20% of lapacho extract, ιC—ι-carrageenan control film, ι5% LE—ι-carrageenan with 5% of lapacho extract, ι10% LE—ι-carrageenan with 10% of lapacho extract, ι20% LE—ι-carrageenan with 20% of lapacho extract.

**Table 7:** The determination of surface and cross-section appearance.

	κC	κ5% LE	κ10% LE	κ20% LE
**surface**	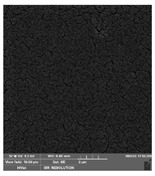	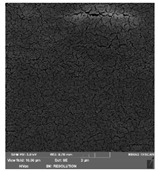	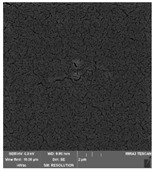	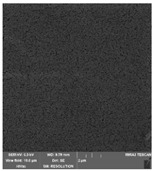
**cross-section**	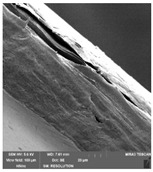	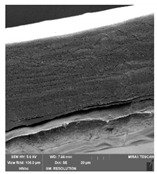	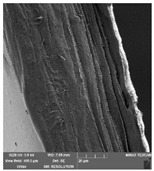	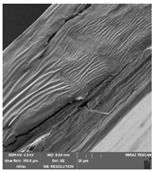
	**ιC**	**ι5% LE**	**ι10% LE**	**ι20% LE**
**surface**	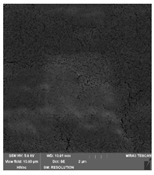	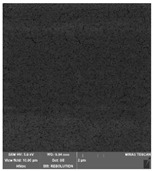	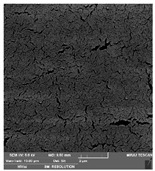	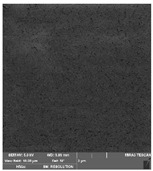
**cross-section**	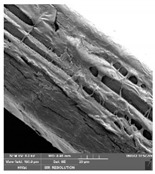	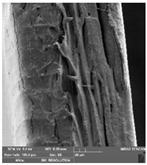	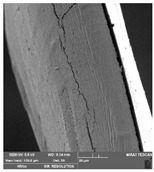	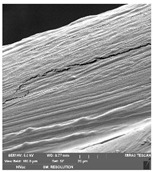

κC–κ-carrageenan control film, κ5% LE—κ-carrageenan with 5% of lapacho extract, κ10% LE—κ-carrageenan with 10% of lapacho extract, κ20% LE—κ-carrageenan with 20% of lapacho extract, ιC—ι-carrageenan control film, ι5% LE—ι-carrageenan with 5% of lapacho extract, ι10% LE—ι-carrageenan with 10% of lapacho extract, ι20% LE—ι-carrageenan with 20% of lapacho extract.
